# Novel Alleles from *Cicer reticulatum* L. for Genetic Improvement of Cultivated Chickpeas Identified through Genome Wide Association Analysis

**DOI:** 10.3390/ijms25010648

**Published:** 2024-01-04

**Authors:** Mohammad Waliur Rahman, Amit A. Deokar, Donna Lindsay, Bunyamin Tar’an

**Affiliations:** Department of Plant Sciences, University of Saskatchewan, Saskatoon, SK S7N 5A8, Canada

**Keywords:** chickpea, genetic diversity, wild accession, population structure, SNP, association

## Abstract

The availability of wild chickpea (*Cicer reticulatum* L.) accessions has the potential to be used for the improvement of important traits in cultivated chickpeas. The main objectives of this study were to evaluate the phenotypic and genetic variations of chickpea progeny derived from interspecific crosses between *C. arietinum* and *C. reticulatum,* and to establish the association between single nucleotide polymorphism (SNP) markers and a series of important agronomic traits in chickpea. A total of 486 lines derived from interspecific crosses between *C. arietinum* (CDC Leader) and 20 accessions of *C. reticulatum* were evaluated at different locations in Saskatchewan, Canada in 2017 and 2018. Significant variations were observed for seed weight per plant, number of seeds per plant, thousand seed weight, and plant biomass. Path coefficient analysis showed significant positive direct effects of the number of seeds per plant, thousand seed weight, and biomass on the total seed weight. Cluster analysis based on the agronomic traits generated six groups that allowed the identification of potential heterotic groups within the interspecific lines for yield improvement and resistance to ascochyta blight disease. Genotyping of the 381 interspecific lines using a modified genotyping by sequencing (tGBS) generated a total of 14,591 SNPs. Neighbour-joining cluster analysis using the SNP data grouped the lines into 20 clusters. The genome wide association analysis identified 51 SNPs that had significant associations with different traits. Several candidate genes associated with early flowering and yield components were identified. The candidate genes and the significant SNP markers associated with different traits have a potential to aid the trait introgression in the breeding program.

## 1. Introduction

The cultivation of improved crop varieties can enhance crop production to achieve food and nutritional security for the growing global population. The role of chickpeas in achieving nutritional security, particularly in developing countries, is crucial due to their high protein, vitamins, and mineral content [1]. The ability of chickpeas to fix nitrogen from the atmosphere plays an important role in maintaining soil fertility and increasing the yield of the succeeding crops in rotation [2]. Chickpeas are traditionally cultivated in the arid and semiarid regions, but the area of cultivation is gradually expanding into other parts of the world including North America. The current average chickpea yield globally is around 1.8 tonnes/ha [3]. Significant yield reduction could occur due to adverse growing conditions like drought, high and cold temperatures, pests, and diseases [4,5,6,7]. There is an opportunity to increase the yield potential of chickpeas up to 5 tonnes/ha by using the diversity available in the crop’s wild relatives, including *Cicer reticulatum* [8,9,10,11]. Genetic improvement of chickpeas mainly targets traits such as yield, abiotic and biotic stress tolerances, plant architecture (upright canopy), early flowering and early maturity, and nutritional qualities through different breeding strategies. Maintaining and increasing genetic diversity is important for crop adaptability in a changing environment. Increasing genetic variation is one of the critical steps for successful crop improvement, as it allows selection to increase or decrease the frequency of certain alleles in the population. A population with high genetic diversity may allow selections of the lines with adaptations to substantially diverse environments.

Crop genetic diversity can be enhanced through introgression of desirable alleles from their wild relatives. The wild species are valuable sources of genes for agronomic traits like early flowering, resistance to biotic and abiotic stresses, and yield potential that can be incorporated into cultivated genotypes [12,13,14]. Interspecific crosses have been implemented as a successful strategy for enhancing the genetic diversity and yield of crops by broadening the genetic base through transferring resistance genes and yield-related alleles from the wild relatives to the cultivated species [10,15]. *Cicer reticulatum*, which is the wild progenitor species of cultivated chickpea, belongs to the primary gene pool of chickpeas along with *Cicer arietinum*, and has the potential to increase genetic variability for seed yield [9,10,16] and other desirable agronomic traits in the cultivated species [17,18,19].

Genetic diversity is usually assessed by using different types of morphological and molecular markers [1,8,20]. Molecular markers have been widely used to determine the genetic variation and the relationship between cultivated crop species and their wild relatives [21,22,23]. Single nucleotide polymorphism (SNP) markers have been used for whole genome scans to reveal the natural allelic diversity in chickpeas [24]. The use of SNPs for genome-wide association study (GWAS) is a promising approach for determining the population structure and genetic dissection of complex traits due its relatively low genotyping cost and high abundance in the plant genome [25]. In this paper, we reported the phenotypic and genetic variation of interspecific chickpea populations derived from crossing *C. arietinum* and *C. reticulatum*. Significant association between SNP markers and a series of important agronomic traits as well as potential candidate genes for early flowering and yield components in chickpeas were identified.

## 2. Results

### 2.1. Variability of Yield and Selected Yield Contributing Traits of Chickpeas

The descriptive statistics revealed a large variation in phenotypic expression of the chickpea interspecific progeny, which could be associated with the genetic variations derived from the wild parents (Table 1 and Table 2). The maximum variance of the mean was obtained for seed weight per plant, number of seeds per plant, and biomass yield per plant (i.e., 75%, 77%, and 99%, respectively, at the Saskatoon site), which was irrespective of different years and sites. For days to flowering and days to maturity, the variances were relatively lower than for other traits. The range and variance of days to flowering and days to maturity were relatively narrow. Ascochyta blight disease caused by the fungus *Ascochyta rabiei* (Pass.) Lab showed a high variability. Some lines were identified as less susceptible to ascochyta with a mean disease score of 4.0. The ascochyta disease infestation in 2018 was lower at Lucky Lake, SK compared to the other sites, which could be associated with the lower prevalence of this pathogen due to limited cultivation of chickpeas and drier conditions in this area. Interestingly, several lines flowered earlier than CDC Leader after 31 days of planting (Table 2), which could be suitable for the short growing season of the Canadian prairies. The highest biomass yield per plant was obtained from some lines evaluated in Saskatoon (Table 1). Growing plants in pots with irrigation and less disease pressure had increased the biomass of the chickpeas. The variability in thousand seed weight was attributed to different seed sizes in the population. In general, the phenotypic variability for traits such as growth habit (50% erect, 40% semi-erect, and 10% prostrate or flat type), seed type (43% desi, 30% kabuli, and 27% pea type) and seed shattering (70% non-shattering and 30% shattering type) were also present within the chickpea lines.

### 2.2. Correlation among the Yield and Yield Contributing Traits of Chickpeas

Correlation analysis was performed among the morphological and yield-contributing traits of the chickpea lines evaluated in two-year field trials (2017 and 2018) at two locations in each year (Table 3, Table 4, Table 5 and Table 6). Among the selected traits, the number of seeds per plant was found to show a significant positive relationship with seed weight per plant at three out of four locations (Table 3, Table 4, Table 5 and Table 6). Biomass was positively correlated with thousand seed weight and number of seeds per plant at all locations. Plant height was positively correlated with the number of primary and secondary branches. Secondary branches per plant also showed a positive correlation with seed yield and number of seeds per plant. Harvest index showed a significant positive correlation with number of seeds and seed weight per plant. However, the relationships of harvest index with days to flowering, days to maturity, plant height, and biomass yield were negative for all sites. Correlation of ascochyta blight disease scores with the harvest index was insignificant in all field trials. Plant height was positively correlated with seed yield across different locations. The relationship of plant height and biomass was also positive. Thousand seed weight had a significant positive correlation with seed yield, whereas it had negative correlation with number of seeds per plant. All the yield contributing traits had a negative correlation with the ascochyta blight disease scores. The relationship between days to flowering and ascochyta blight disease was also negative in the 2018 field trials.

### 2.3. Path Coefficient Analysis

The structural equation model (SEM) was constructed to determine the direct and indirect effects of the yield contributing traits on the seed yield of chickpeas (Table 7 and Table 8). The SEM statistical approach or path analysis was used to quantify the causal relationships among the selected intercorrelated traits. Results from the path analysis revealed that the number of seeds per plant had the highest direct positive effect on seed weight per plant, followed by thousand seed weight. These traits consistently showed positive effects on seed weight per plant in three out of four of the environmental conditions of different field sites. Therefore, these traits are potential objects for selection in the breeding program to increase chickpea yield. The direct effect of thousand seed weight on the number of seeds per plant was negative. The biomass also had a negative direct effect on harvest index. However, the indirect effect of biomass on the harvest index was positive in one out of four locations. Among all the traits, the number of seeds per plant and seed weight per plant showed a positive direct effect on the harvest index. The direct effect of thousand seed weight on harvest index was positive in three out of four environments.

### 2.4. Effects of Genotype, Environment and their Interaction on Seed Yield and Yield Contributing Traits of Chickpeas

Plant growth and seed yields of chickpeas were greatly influenced by genetic and environmental factors. The analysis of variance (ANOVA) using mixed linear model revealed significant effects of genotype (G) and environments (E) for all of the traits (Table 9). The G × E interaction components were significant for days to maturity, number of seeds per plant, thousand seed weight, and seed yield, whereas their interaction effects on days to flowering, plant height, and biomass were not significant (Table 9). Broad-sense heritability estimates (*H*^2^) showed low-to-medium heritability for all traits (Table 9). The maximum *H*^2^ was observed for days to flowering (0.54) followed by seed weight per plant (0.45) and days to maturity (0.35). The yield contributing traits, such as number of seeds and biomass yield per plant had *H*^2^ of 0.18 and 0.14, respectively. Thousand seed weight had the lowest *H*^2^.

### 2.5. Cluster Analysis Based on Agronomic and Yield Traits

**Figure 1:**
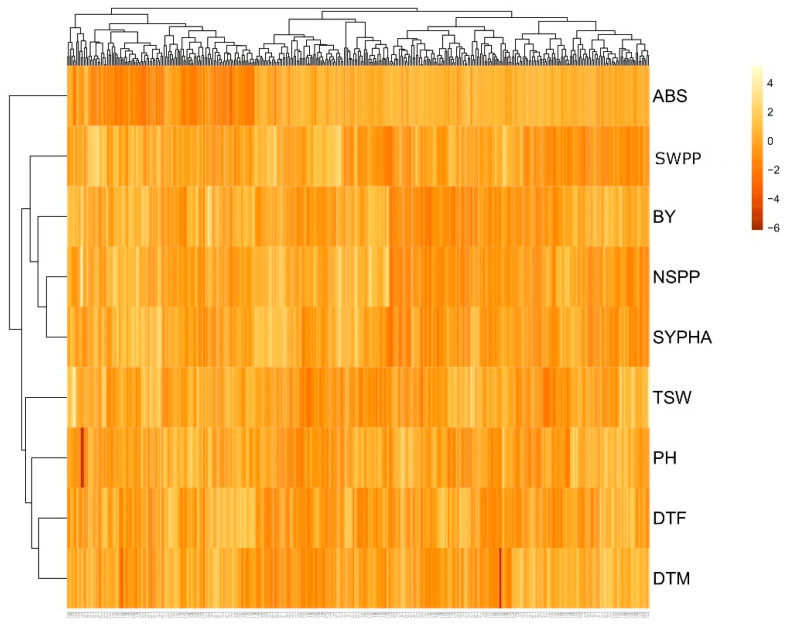
Heatmap based on the agronomic and yield components summarizing the differentiation among the 381 F_5_’ lines following the Euclidean Ward method. Different traits are DTF: days to flowering; DTM: days to maturity; PH: plant height (cm); TSW: thousand seed weight (g); SYPHA: seed yield per hectare (kg/ha); NSPP: number of seeds per plant; BY: biomass yield per plant (g); SWPP: seed weight per plant (g); ABS: ascochyta blight disease score.

The standardized mean values of the nine agronomic traits from the 381 F_5_’ lines were used in cluster analysis (Figure 1). The means and standard deviations of six major clusters offered information regarding the genetic diversity of the lines. They provided an opportunity to identify the best group (i.e., cluster I = 67 F_5_’ lines), which possessed high seed yield and a combination of desirable agronomic traits. The largest group belongs to cluster II (104 F_5_’ lines), while cluster VI had the lowest number of lines (28 F_5_’ lines). Cluster I produced the highest number of seeds per plant and seed weight per plant which were comparatively higher than the other clusters (Table 10). The cluster I lines could be recommended for future breeding programs to improve the yield of chickpea. Lines in cluster VI contained several important traits, including early flowering and early maturity with the mean values of 45 and 86 days, respectively (Table 10). Cluster VI also showed the lowest ascochyta blight disease score, indicating less susceptibility to the disease. As such, cluster VI could be a potential source for further improvement of ascochyta blight resistance in the breeding program.

### 2.6. Genetic Diversity and Population Structure Analyses

**Figure 2:**
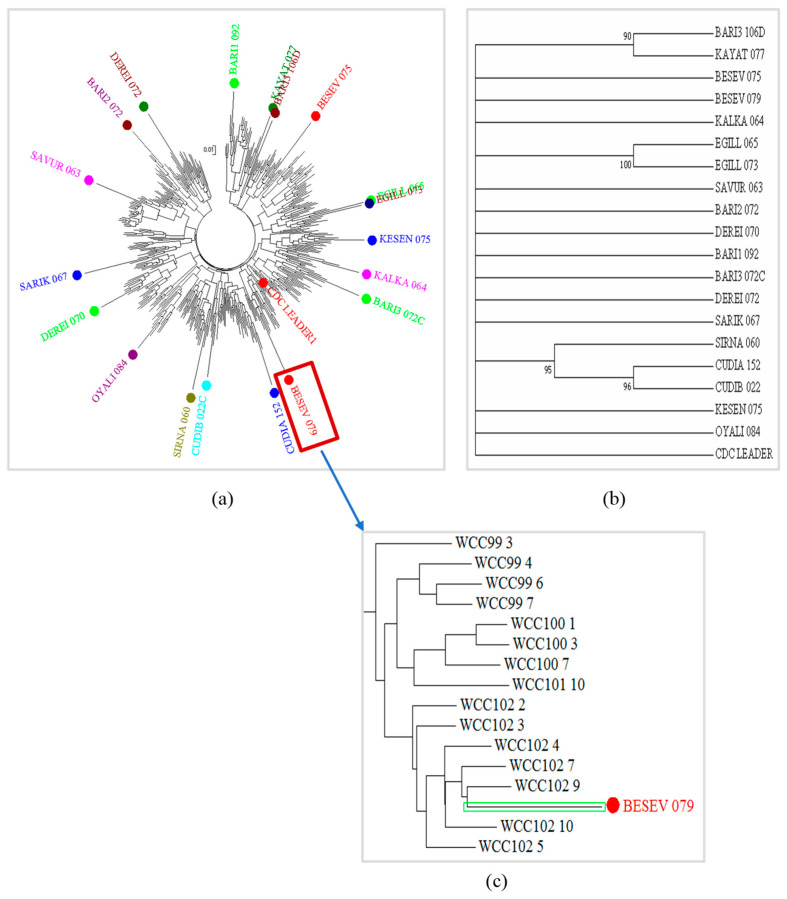
(**a**) Neighbour-joining (NJ) clustering revealed the genetic relationships of 381 chickpea lines including 19 wild and 1 cultivated parent using 14,591 SNPs markers with MAF ≥ 1%. The colour dots indicate different parents that were crossed with the cultivated parent (CDC leader) to develop the chickpea lines. (**b**) Phylogenetic tree and bootstrap values of 19 wild and 1 cultivated parent (CDC Leader) were developed based on the SNP markers. (**c**) Visualization of the formation of clusters of the chickpea lines with their respective parent.

The genetic diversity of 381 F_5_’ lines, the 19 wild parents, and 1 cultivated parent (CDC Leader) was analyzed following NJ tree clustering using MEGA X program. Genotyping of the population by tGBS (a modified genotyping by sequencing method) identified a total of 15,186 SNP markers. After filtering with MAF = 1%, the number of SNPs were reduced to 14,591 and used to calculate the genetic diversity of the chickpea lines. The distribution of SNP markers on the chromosomes indicated the highest number of SNPs on chromosome four. This implies that chromosome four might have a greater contribution towards the diversity of the chickpea population. The NJ cluster analysis divided the 381 F_5_’ lines into 20 distinct groups according to their respective cultivated and wild parents from which they were developed (Figure 2a). It was expected that some useful genetic information in the respective wild parents were transferred to the progeny lines. Further, the clustering patterns of the 381 F_5_’ lines were consistent with their wild parents (Figure 2c). The diversity analysis of the cultivated and wild parents produced 16 different clusters (Figure 2b).

**Figure 3:**
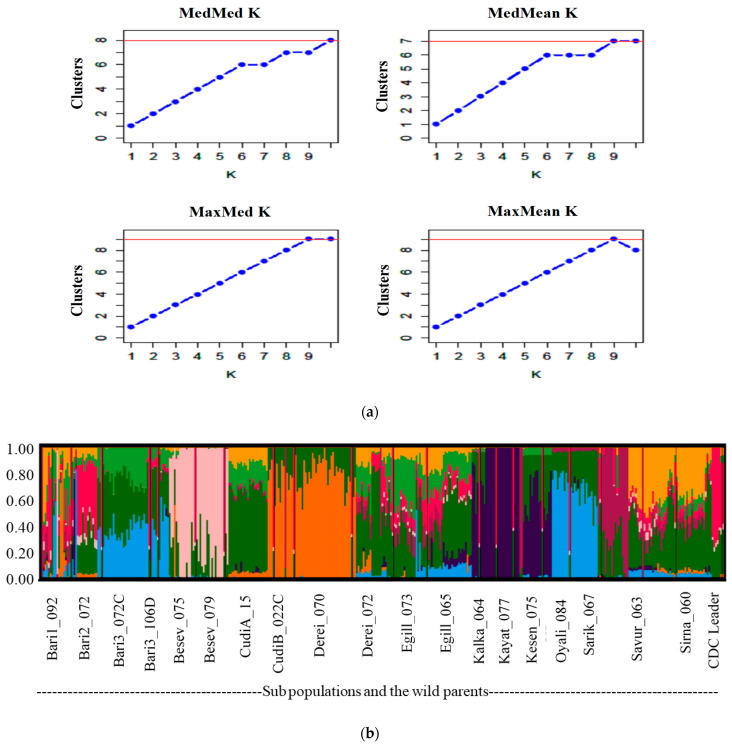
Admixture analysis of the 381 chickpea F_5_ lines with their 19 wild parents and 1 cultivated parent (CDC Leader) was performed with *K* = 2 to 10 based on the polymorphic markers. The individual line was represented by a thin vertical line and the colour-coded admixture proportions indicate the genetic contributions of the parents. (**a**) Identification of the number of clusters of 401 chickpea lines. The blue line indicates the mean and median values, whereas the red line indicates the most likely number of genetic clusters (*K* = 9). (**b**) Visualization of the chickpea population clusters as revealed by ADMIXTURE analysis. When *K* = 9, the population was classified into nine groups.

To provide further insights into the genetic diversity, the population structure was determined using the ADMIXTURE analysis. In this analysis, the 14,591 SNPs with MAF ≥1%, and the number *K* from 2 to 10 (repeating each analysis 20 times) were used to find the best *K* peak. The highest peak value was observed at *K* = 9, which indicated the possibilities of the presence of 9 clusters within the 381 lines (Figure 3a). As the curve became plateaued or started to decline at *K* = 9 (Figure 3a), it provided a strong support to form 9 clusters from the lines. Furthermore, the ADMIXTURE analysis revealed some degrees of intermixing of the lines in each cluster. Thus, the sample lines could be considered as weakly differentiated. However, the population structure as shown in Figure 3b indicated that the lines developed from Besev_075 and Besev_079, as well as Egill_073 and Egill_065, were clustered together, and these two clusters were clearly distinct from other groups. The formation of the two subpopulations derived from these four parents could be associated with the variability of the geographical conditions where these accessions were collected.

### 2.7. Association Analysis and Potential Candidate Genes

**Figure 4:**
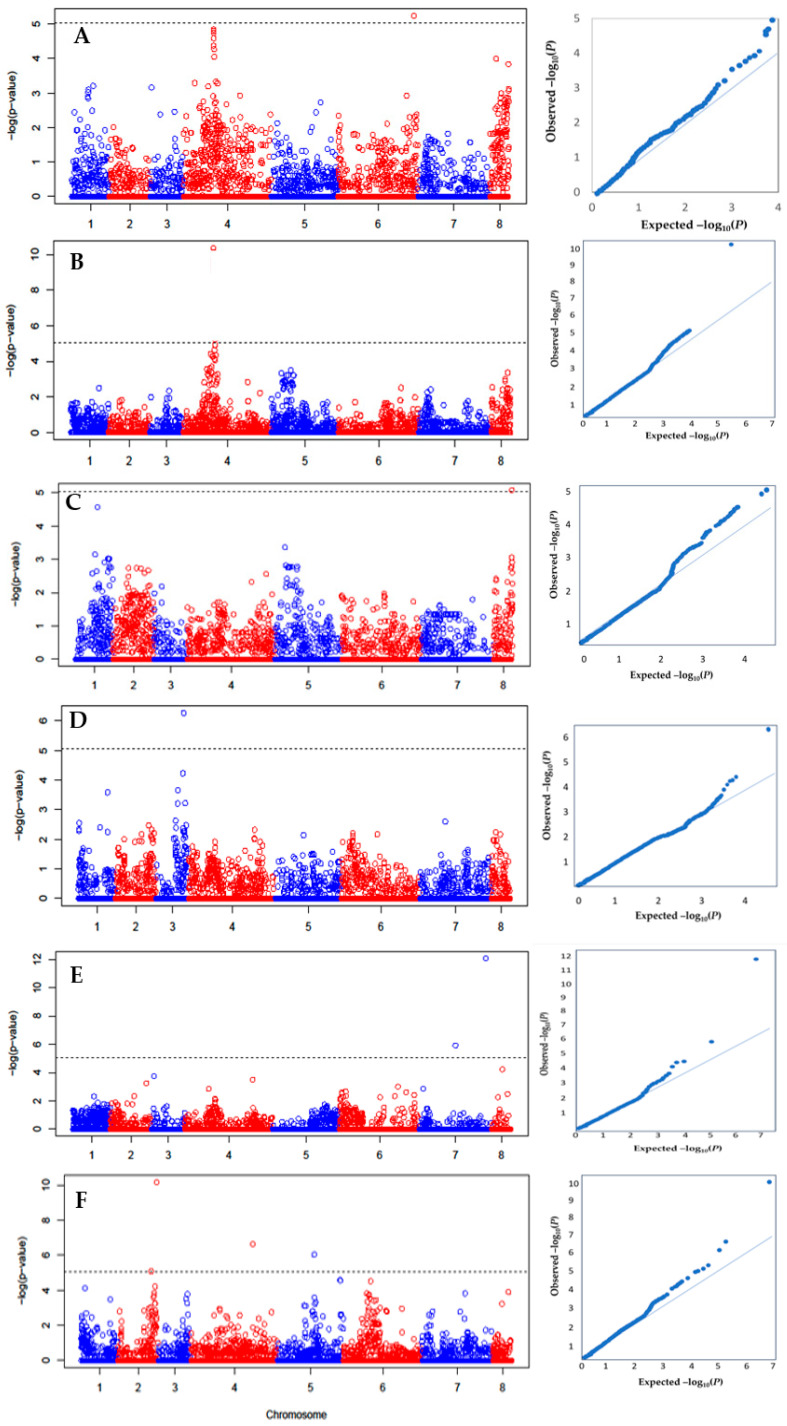

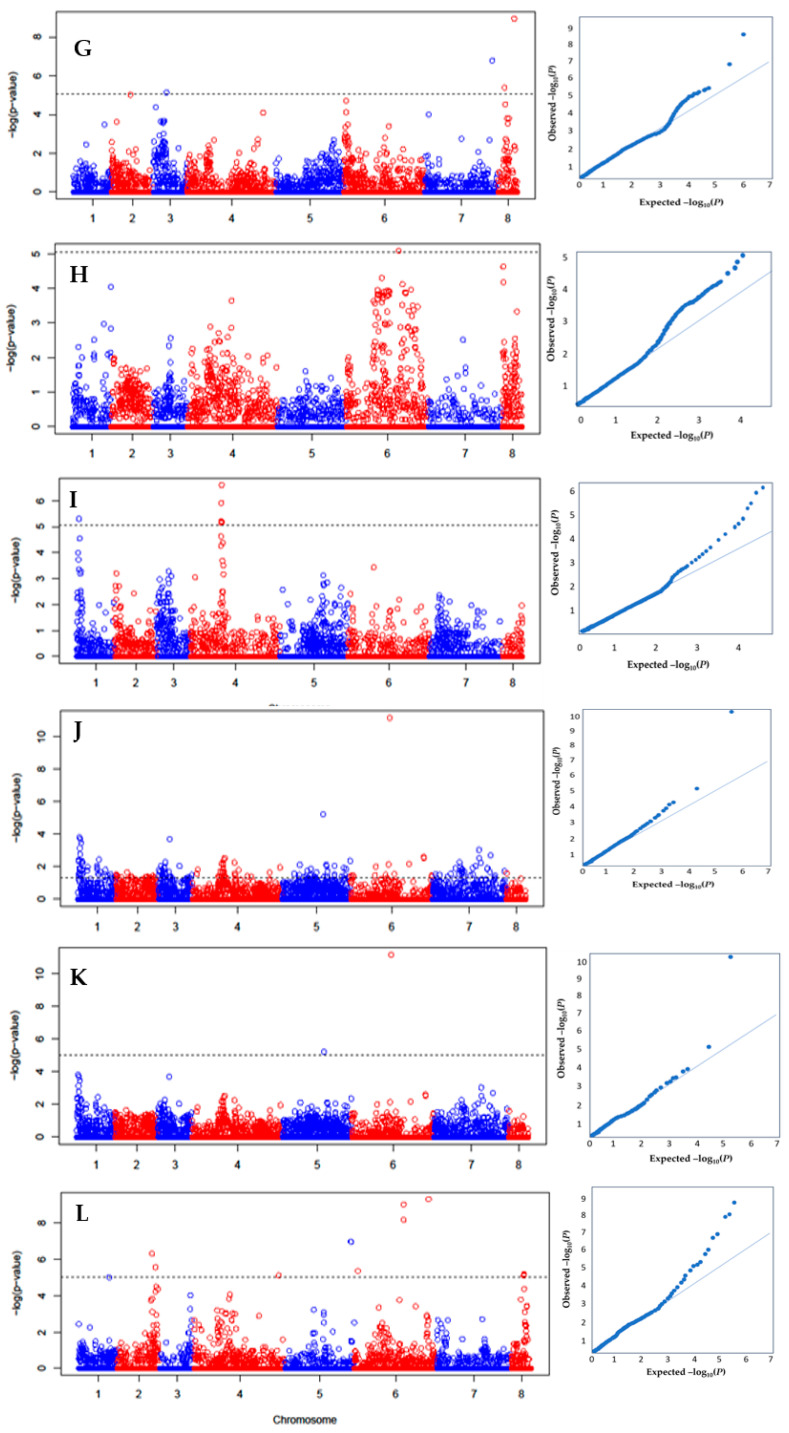
Genome-wide association analysis −log (*p*) value for marker association and its corresponding Q–Q plot for: (**A**) days to flowering (Combined-2017), (**B**) days to flowering (Combined-2018), (**C**) days to maturity (Lucky Lake-2018), (**D**) biomass yield (Combined-2017), (**E**) biomass yield (Combined-2018), (**F**) number of seeds per plant (Saskatoon-2017), (**G**) number of seeds per plant (Moose Jaw-2017), (**H**) number of seeds per plant (Lucky Lake-2018), (**I**) thousand seed weight (Moose Jaw-2017), (**J**) thousand seed weight (Limerick-2018), (**K**) thousand seed weight (Lucky Lake-2018), (**L**) seed weight per plant (Saskatoon-2017).

The association between the SNPs and the agronomic traits were calculated using 5501 SNPs that have MAF ≥ 5% and the mean values of the agronomic and yield contributing traits obtained from the field evaluation of 381 F_5_ lines (Figure 4A–L). After filtering with MAF ≥ 5%, the scaffolds were removed in order to exclude the potential redundant markers. There were 51 SNPs identified on different chromosomes which showed significant association with 5 traits such as days to flowering, biomass yield (g), thousand seed weight (g), number of seeds per plant, and seed weight per plant (g) (Table 11 and Table 12).

A SNP locus (Ca4_V1_P-13022400) within the 13.0 Mbp region on chr4 was responsible for early flowering in the genotypic panel (Figure 4A). Further analysis confirmed that the alleles from the wild parents were responsible for the early flowering. The highly significant SNP marker (Ca4_V1_P-13022400) was identified using the combined data of 2018 field sites which was associated with days to flowering and explained 0.3% to 5.0% phenotypic variance (R^2^) for this trait. There was no SNP significantly associated with plant height. The number of SNPs significantly associated with different traits were: 1 SNP for days to maturity (R^2^ = 12%), 3 SNPs for biomass yield (R^2^ = 1.0 to 6.0%), 13 SNPs for number of seeds per plant (R^2^ = 0.4 to 1.1%), 12 SNPs for thousand seed weight (R^2^ = 0.2 to 3.0%), and 13 SNPs for seed weight per plant (R^2^ = 0.1 to 32%). The highest mean difference between wild and cultivated alleles for these traits were 3.0 days for days to maturity, 8.5 g for biomass, 4.0 for number of seeds per plant, 16.0 g thousand seed weight, and 4.3 g seed weight per plant, respectively (Table 11 and Table 12).

Potential candidate gene identification was performed within 100 Kb region on either side of the significant markers via legume information system (LIS). Seven candidate genes were found in the regions, three of which are related to flowering and four of which are related to the growth, development, and yield (Table 13).

## 3. Discussion

The narrow genetic base of cultivated chickpea germplasm is restricting the opportunities of genetic advancement for higher yield, quality, and desired agronomic traits. Several studies [24,32,33] reported that the valuable genes that were lost through the domestication and recurrent selection process could have a significant contribution in the development of new varieties with higher yield, quality, and increased tolerance to biotic and abiotic stresses. Conversely, the wild relatives of chickpeas are considered the most significant sources of genetic variability, and have promising potential for variety improvement [1,18]. *C. reticulatum* is considered as one of the most important wild species closely related to cultivated *C. arietinum,* and exhibits a high cross-compatibility [18,34,35]. This ultimately provides an opportunity to successfully utilize the potential advantage of cross-compatibility between *C. reticulatum* and *C. arietinum* for developing interspecific hybrids of chickpeas [10,36].

### 3.1. Variability and Performance of the Interspecific Lines

The segregating populations used in this research were developed from interspecific crosses between twenty accessions of *C. reticulatum* and one cultivated (CDC Leader) parent. Two successive generations (F_4_ and F_5_) of chickpea lines derived from the interspecific crosses were evaluated at four locations in Saskatchewan. The populations were completely fertile and capable of producing fertile progenies. The population revealed a considerable variation for seed yield and agronomic traits (Table 1 and Table 2). Our results were in line with the previous findings [35].

The wild accessions used in this research were known to have high genetic variation [37] and contributed to improving the productivity as well as resistance to biotic and abiotic stresses under the environmental conditions of California, USA [37]. The initial anticipation of genetic variability in these wild germplasms is based on their diverse geographical distribution and adaptability to varying environmental conditions. The variability in the current population is described by using the mean, range, and variance of the means of a specific trait (Table 1 and Table 2). The results showed considerable variations among the studied lines for all the yield-contributing and agronomic traits. Different yield-contributing characteristics such as seed weight per plant, number of seeds per plant, and biomass yield per plant showed the maximum variability. The large variation in yield in the progeny lines suggested that the favourable genes have been transferred by interspecific crossing. In many instances, the segregating lines developed from the interspecific crosses showed high genetic variability for different traits including the number of branches per plant and harvest index [10,35,38]. Both genetic and environmental factors, as well as the interactions between genes and environmental factors, might have contributed to this type of observed variation. A recent study of chickpeas grown at eight different locations in Australia showed a significant influence of the environment on the genetic variation for yield [39]. The large yield variation is typically due to the introgression of genes from the wild *Cicer* species [35,40]. Similar findings on the increased phenotypic variability in the cultivated chickpeas that was derived from interspecific crosses between wild and cultivated variety were reported in India [9,35]. The variation observed for the growth habit such as erect and semi-erect types of plants can facilitate a potential advantage for mechanical harvesting. Thus, proportions of lines with preferred qualitative traits listed can offer to identify some erect or semi-erect genotypes suitable for mechanized agriculture of the Canadian prairies. Moreover, the variation in seed types such as kabuli, desi, and round or pea type could be utilized for further development of varieties to satisfy consumer demand. Overall, the genotypes with erect and semi-erect characteristics that exhibited less susceptibility to diseases, better harvest index, and high seed yield could be utilized for the development of commercially acceptable chickpea varieties for growers in western Canada.

### 3.2. Interrelationships among the Yield and Yield Contributing Traits for Efficient Selection

Extensive knowledge of genetic variability and relationships among the yield contributing traits can easily justify the success and effectiveness of breeding strategies. Usually, seed yield is considered as a complex trait, and is profoundly influenced by all agronomic and yield-contributing characteristics. Correlation analysis is one of the most common approaches for evaluating the relationships among traits and to identify the most important ones contributing to seed yield [41]. The relationships among various traits are useful for selecting genotypes with higher productivity based on groups of desired traits that significantly contribute to the increased yield of chickpeas.

The use of correlation statistics is well documented for genotype selection in variety improvement programs [41,42,43]. However, the selection strategies could be effective only when the traits exhibit a significant and positive correlation with seed yield. For instance, the high yielding chickpea genotype selections were performed in a study conducted in Pakistan depending on the traits that showed a significant and positive correlation with yield [44]. The positive correlation was between the number of seeds per plant and seed yield observed in this study, suggesting that the increase of number of seeds per plant could be an effective way to increase the chickpea yield under the western Canadian soil-climatic condition. Similarly, positive correlation of total seed yield with biomass yield and harvest index was also observed in earlier studies [45,46]. These traits were used for further breeding to increase chickpea seed yield [45]. The biomass yield showed a significantly positive correlation with the number of seeds per plant in three out of four locations over two years. Path analysis also confirmed this finding and indicated that the biomass yield has a significant direct effect on the number of seeds per plant. Therefore, the selection of genotypes with higher biomass could be a potential option to improve the seed yield of chickpeas. Path coefficient analysis also confirmed the significant and high positive direct effects of other yield components such as thousand seed weight on the total seed weight per plant in three out of four locations over two years.

The thousand seed weight was negatively correlated with the number of seeds in 2018 field trials, which agreed with previous research findings [46]. Apart from plant height, other traits such as the number of days to flowering and ascochyta blight disease infestation showed negative relationships with seed yield at one out of four locations, and the relationship between days to maturity and seed yield was inconsistent. The research conducted earlier under tropical weather conditions revealed that the days to flowering and days to maturity had a negative correlation with seed yield [47]. Severe yield loss of chickpea with the increase of ascochyta blight disease was also observed [48,49]. Conversely, previous research reported that chickpea yield is mostly affected by the environment, and the yield performance of a variety was inconsistent under different environmental conditions of the Canadian prairies [50].

### 3.3. Genotype by Environment Interaction, and Broad Sense Heritability

Evaluation of the chickpea lines under two different environmental conditions provided an opportunity to select stable genotypes that could be useful for further development of breeding populations with higher adaptability in the changing environment [39,51]. Generally, the seed yield is a complex trait, controlled by multiple genes, and strongly influenced by the interaction between the environmental factors and yield contributing traits [52,53]. The most significant genotype by environment interaction effects were observed for days to maturity, the number of seeds per plant, thousand seed weight, and seed yield. Therefore, better documentation on the genomic approach for the perception and processing of environmental signals need to be developed. Several researchers [39,54,55,56] have evaluated the influence of environment on the yield components of different legumes and selected the identical genotypes with improved yield under varying environmental conditions. However, chickpea yields are highly influenced by genotype and environment interactions, and exhibit poor heritability under marginal and unfavourable environments [52,53]. Overall, the interaction between genotypes and the environment for economically important traits deserves further attention.

The broad-sense heritability estimated from the variance components resulted in low-to-moderate heritability for the traits in the current study. The highest heritability was observed for days to flowering, which indicated that this trait is favourable for the selection of better-performed chickpea lines under the Canadian prairie conditions. A similar finding on high heritability for days to flowering was reported in research conducted with 47 chickpea genotypes in Pakistan [57]. Using the knowledge of the heritability for the selection of the best progenies is crucial for better transmissibility of traits in variety improvement programs [58,59,60]. The low-to-medium *H^2^* expressed by the traits in the current study was associated with the significant effects of the environments on the phenotypic expression. The low heritability for the yield components observed in this study agreed with the previous research findings [47,61]. In general, the heritability of a specific trait changed over time due to variation of temperature and environmental conditions [52,53].

### 3.4. Clustering of the Chickpea Lines Based on the Phenotypic Traits

Cluster analysis using the phenotypic traits can be used to separate the genotypes into distinct groups based on a particular trait of interest [62]. In this study, the Euclidean distance following Ward’s method was used to identify six clusters. The result also indicated the morphological diversity of the interspecific lines. The grouping of 381 chickpea lines into those clusters was based on similarity matrix, therefore, it was considered a completely random process as no relationship between pedigree and genetic diversity was observed. Furthermore, a given cluster included some diverse lines that were developed from different parents. Among the six groups, the cluster I comprised 67 lines with important yield traits such as the seed weight per plant, the maximum number of seeds per plant, and the highest total seed yield. The cluster IV consisted of 72 lines which have a high thousand seed weight. The lowest number of lines (28) were found in cluster VI, which was categorized with early flowering, early maturity, and reduced susceptibility to ascochyta blight disease. These observations suggested the possibility of yield improvement by combining high seed yield with increased seed weight through effective selection and hybridization between the genotypes of cluster I and cluster IV. Previous research suggested the possibility of attaining hybrid vigour from crossing between genotypes of distant clusters [63]. Furthermore, the hybridization between genotypes of cluster I and cluster VI will facilitate the opportunity to incorporate the commercially demanding traits of prairies (such as early flowering, early maturity, and reduced ascochyta susceptibility) in high yielding genotypes.

Many researchers reported the clustering of chickpea genotypes through a similarity matrix to evaluate the phenotypic diversity for desirable traits and successfully identified the most diverse genotypic groups [64,65]. However, the clustering pattern of the chickpea lines was irrespective of their parental germplasms. Additionally, the lines derived from crossing between parents were found to group into different clusters. A similar clustering pattern was used to evaluate the genetic diversity of chickpea genotypes based on the highest performance in desired agronomic and yield traits [44,64].

### 3.5. Genetic Diversity in the Chickpea Lines Using SNPs

Effective utilization of plant genetic resources for variety improvement largely depends on the available genetic diversity of the breeding population. Previous research reported that a better understanding of genetic diversity in chickpea germplasms can contribute to the selecting and adopting of novel breeding strategies for superior variety development [1]. Usually, the natural allelic variation in the wild *Cicer* is much higher than the cultivated species [66], therefore, the wild *Cicer* species were considered as a potential source of desirable genes for commercially valuable traits [1,67]. The introgression of desirable alleles from wild species through interspecific hybridization is considered the best approach for improving the genetic variation in cultivated chickpeas [67,68]. Some studies [10,69] showed successful breeding with wild *Cicer* species to increase the genetic diversity of chickpeas. The genetic diversity and population structure results of this study also confirmed a substantial amount of genetic variation in the developed breeding lines.

Recent improvements in genotyping-by-sequencing have led to the generating of a large number of cost-effective genome-wide molecular markers such as SNPs [70,71,72]. Additionally, the molecular marker technology is being used widely in chickpea breeding programs to investigate the diversity, genetic relationship, and marker-trait association due to their tight linkage with important agronomic and adaptive traits [1,49,73]. Several researchers [21,72,74,75] reported abundant SNP markers throughout the genome and their effective association with genes controlling a specific trait. Therefore, SNPs are used for estimating the genetic diversity, population structure, and marker-trait associations which are essential for evaluating the genetic potential of the experimental germplasms.

Different approaches (i.e., neighbour-joining and admixture) [76,77] were used in this study which were known to give better indications of genetic diversity and the structure of the studied chickpea lines. Also, the studied lines were genotyped by a modified genotyping-by-sequencing method called tGBS using two restriction enzymes (NspI and BfuCI) [78]. This method used single-stranded oligos instead of double-stranded adaptors that simplified the tGBS library preparation [78]. Moreover, it is well suited for genotyping germplasms with available reference genomes, showing high SNPs calling accuracy, and generating less missing data per site. In this study, the SNPs identification was performed using the reference genome of CDC Frontier (Version 1.0) [24]. The SNP markers generated by tGBS method were used to determine the genetic relationship among the 401 chickpea genotypes by the neighbour-joining method. This method was used extensively to explain the evolutionary relationships among the diverse crop genotypes [79,80]. Increased diversity in the chickpea germplasms is evident from the grouping of breeding lines with their respective wild parents used in this crossing program. The information derived from diversity analysis could be utilized for developing cultivars with desirable agronomic traits through crossing between genotypes from a different cluster. The SNPs were found capable of explaining the reason for the clustering of some lines and irrespective parents as greater similarities with other progenies were identified. Additionally, the genetic relationship analysis in the parents indicated the formation of 16 groups out of 19 wild, and 1 cultivated parent used in crossing. However, the lack of distinct differentiation in wild and cultivated accessions were also reported in some recent studies [80,81] and likely to be associated with the low genome coverage sequencing [82]. These specific or isolated groups could be associated with the adaptability, growth pattern of the wild parents, and the environmental conditions of the areas from which they were collected. The wild parents were collected from different elevation gradients. These accessions may possess useful genetic variation for adaptability and seed quality. For example, the Sirna_060 parent was collected from the highest elevation (1658.92 m) which had distinct environmental conditions such as low temperature in winter and high annual rainfall [37]. The results agreed with similar studies conducted previously [37,83] which reported the presence of large diversity in the wild accessions collected from the similar regions of Fertile Crescent and successfully utilized those wild parents for improving the genetic diversity in chickpea.

Admixture analysis using SNPs is essential for determining the genetic structure of introgressed lines with important agronomic and yield contributing traits [77,82]. These results indicated that the SNP markers categorized the lines into nine groups (*K* = 9) along with a little intermixing of lines in them. However, it is not unusual to exhibit admixed ancestry traces in the developed breeding lines as has been reported in different studies [82,84,85]. Furthermore, the phylogenetic relationship analysis also supports the formation of groups with admixed chickpea lines derived from different parental crosses. Typically, the presence of admixed ancestry in populations was likely related to the wild parental accessions that inherited similar gene pools [77,80]. In a recent study [82], it was confirmed that admixture analysis was capable of identifying the relationships between the breeding population with their ancestry that could be utilized for marker-assisted breeding programs of chickpeas. Finally, the genetic diversity and population structure revealed in this study could be used in future breeding efforts to improve chickpeas.

### 3.6. Association Mapping of the Studied Traits

The use of association mapping is considered as a powerful tool to identify the SNP markers associated with important agronomic traits [79]. In the past, several studies were known to use SNP markers to depict marker-trait associations in segregating population evaluations for new variety development of chickpeas [82,86,87,88]. The identification of the molecular markers showed that the genes governing yield and agronomic traits are widely distributed throughout the genomic region of chickpea [24]. Therefore, the identification of markers associated with the candidate genes that govern novel agronomic traits is vital for variety improvement.

Our association analysis integrated the phenotypic data of 381 chickpea lines with the genotypic information to identify the SNPs associated with the commercially acceptable traits. All phenotypic data obtained from the field study have been used for association mapping, however, four traits have shown significant variation under two different environmental conditions. Based on the physical position of the SNP markers, several genes associated with flowering and yield-related traits were identified. The presence of candidate genes on different chromosomes [82,89] are closely matched with the locations of the current significant makers. The significant SNPs were found on chromosomes four and six and showed a relationship with the flowering time of chickpeas. These results agreed with earlier studies that reported the presence of markers on similar chromosomes (i.e., four and six), and significantly associated with the flowering time of chickpeas [53,89,90,91]. Additionally, the markers identified for yield and yield-contributing traits such as days to maturity, biomass (g), number of seeds per plant, thousand seed weight (g), and seed weight per plant (g) of this study are distributed widely on different chromosomes. Similar results were reported earlier [40] that identified that the SNP loci associated with seed yield are widely distributed throughout the genomic regions of chickpea. The genetic basis of the protein produced by the flowering related genes have been well characterized in *Arabidopsis thaliana* [26,28,92]. The identified genes involved in encoding kinase protein, plastocyanin, and PsaG/PsaK protein are known to be associated with the seed yield trait in chickpeas as it controls the molecular pathways of underlying growth, development, and yield traits [27,30,31]. Overall, seed yield is considered as a complex quantitative trait and the continuous marker-assisted breeding research has identified numerous genomic regions that can govern crop yield [29,38,51,93].

## 4. Materials and Methods

### 4.1. Source of Germplasm

**Figure 5:**
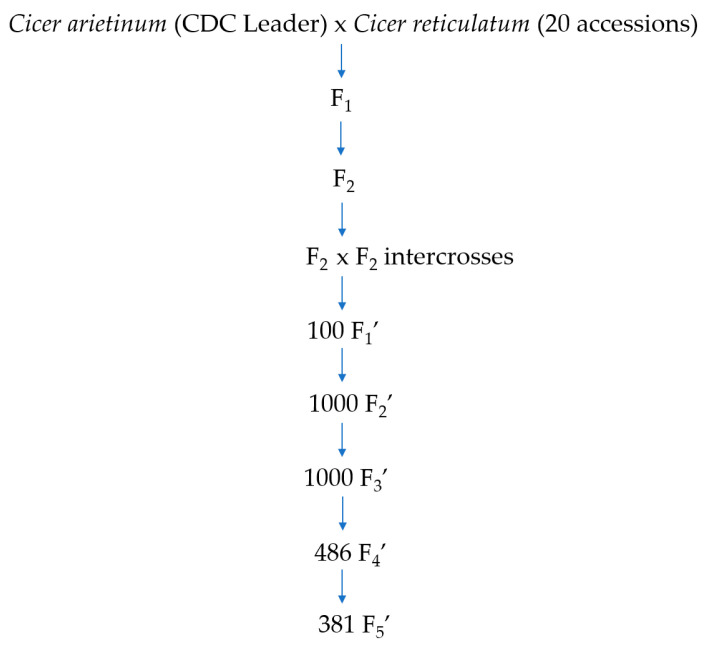
Flow diagram of the development of interspecific chickpea lines.

The research was conducted at the University of Saskatchewan as a part of the chickpea improvement program in which 20 accessions of *C. reticulatum* were used in crosses to develop the interspecific progeny of chickpeas (Figure 5). The wild species parents were part of the project of the Chickpea Innovation Lab led by Dr. Doug Cook at the University of California, Davis, USA. The wild germplasms were collected from diverse geographical locations in Turkey, which varied significantly in terms of phenology and resistance to biotic and abiotic stresses [37]. The wild accessions of *C. reticulatum* have purple flowers, and seeds with distinct shapes and colours. The cultivated variety CDC Leader is a kabuli type with high-yield potential, white flower colour, and beige colour seeds with typical ram-head shape. This variety can attain 42 cm of plant height and has moderate resistance to ascochyta blight disease.

### 4.2. Development of Chickpea Lines from Interspecific Crosses of C. arietinum x C. reticulatum

In the summer of 2014, the initial crosses were made between the adapted cultivar (CDC Leader) and 20 wild accessions of *C. reticulatum* under greenhouse conditions at the University of Saskatchewan. The F_1_ seeds were grown, and each F_1_ plant was cloned through the process of stem cutting to maximize the production of F_2_ seeds [94]. The F_2_ plants with white flower and purple flower within each population were intercrossed to increase the diversity within each population. A total of 1000 F_2_’ were developed from selfing 100 F_1_’ from the last crossing. The F_3_’ (1000 lines) were grown under field conditions in Limerick, Saskatchewan in the summer of 2016. Due to high ascochyta blight disease pressure, only 486 F_3_ lines survived and produced sufficient F_4_’ seeds. The 486 F_4_’ were grown in Moose Jaw and Saskatoon, Saskatchewan in the summer of 2017. In the following year (summer 2018) the remaining 381 F_5_’ were grown in Lucky Lake and Limerick, Saskatchewan (Figure 5).

### 4.3. Experimental Setup, Data Collection, and Management

Field experiments to assess the variations of the F_4_’ and F_5_’ lines were conducted in the 2017 and 2018 growing seasons, respectively, at two locations in Saskatchewan in each year. In 2017, the field sites were in Saskatoon (52°07′27.2″ N and 106°36′47.4″ W) and Moose Jaw (50°01′16.0″ N and 106°20′30.7″ W). In 2018, the experimental field sites were in Lucky Lake (51°3′57.94″ N and 107°11′34.74″ W) and Limerick (49°38′28.12″ N and 106°29′15.91″ W) Saskatchewan, Canada. The experimental sites were located in the brown (Lucky Lake and Limerick) and dark brown (Saskatoon and Moose Jaw) soil-climatic zones of Saskatchewan. The individual plot size was 1 m × 1 m. On average, 42 seeds were planted in 3 rows per plot. Prior to seeding, all seeds were treated with Insure^®^ fungicide (Triticonazole, Metalaxyl, and Pyraclostrobin) as recommended for pulse cultivation.

In 2017, 486 F_4_’ lines were evaluated in Moose Jaw with a single replication due to the limited amount of seeds. Multiple checks were used in the experiment. Simultaneously, each of the 486 F_4_’ lines and the checks were grown in two-gallon plastic pots in the yard of the Crop Science Field Lab at the University of Saskatchewan campus in Saskatoon. These two experiments were laid out as modified augmented designs (MADs), where the checks were replicated three times [95]. In this study, eight chickpea varieties developed at the Crop Development Centre were used as checks, such as CDC Leader, CDC Frontier, CDC Palmer, CDC Orion, CDC Alma, CDC Corinne, CDC Cory, and CDC Consul. At the Moose Jaw field site, selection was conducted based on the ascochyta blight disease infestation. Lines that had a very high ascochyta blight disease score (8 or higher on a 1–9 rating scale) and produced no or a limited number of seeds were eliminated for the next generation trial. In 2018, 381 selected F_2:5_ lines were evaluated in Lucky Lake and Limerick, SK. In these sites, the experimental design was a randomized complete block design (RCBD) with three replications. In 2017, seeds were sown on May 11th and May 23rd at the Moose Jaw and Saskatoon sites, respectively. In 2018, the seeding dates for Lucky Lake and Limerick field sites were May 3rd and May 7th, respectively. Only nitrogen fertilizer was applied as side band during seeding at all the field sites. No rhizobial inoculant was applied. Fungicide (Priaxor) and herbicide (spring burn-off Roundup, Clethodim, Amigo, and Axial) were applied for disease and weed control.

Data were collected for agronomic and yield traits including plant height, days to flowering, days to maturity, number of primary and secondary branches, ascochyta blight disease rating, growth habit, seed type, seed shattering, biomass per plant, number of seeds, seed weight per plant, and thousand seed weight. Three randomly selected plants (2018 field trial) and six randomly selected plants (2017 field trial) from each plot were harvested by hand to estimate the plant biomass and yield traits. The flowering, maturity, and growth habit data were recorded on individual plot basis. Plant height of each plot at all the experimental sites was recorded at the maturity stage. Three plants were randomly selected from each microplot for plant height, which was measured from the ground level prior to harvesting. The branching pattern was used to categorize the plant architecture as erect, semi-erect, and prostrate type of growth habit. The ascochyta disease score was conducted on the plot basis during pod formation period. The rating and scoring of ascochyta blight disease was performed by visual observation using 1 to 9 rating scale such as 1 = healthy plant, no disease; 2 = lesions present, but small and inconspicuous; 3 = lesions easily seen, but plant is mostly green; 4 = severe lesions clearly visible; 5 = lesions girdle stems, most leaves show lesions; 6 = plant collapsing, tips die back; 7 = plant dying, but at least three green leaves present; 8 = nearly dead plant (virtually no green leaves left) but still with a green stem; and 9 = dead plant (almost no green parts visible) [96]. Days to flowering was recorded as the number of days from sowing to the stage when 50% plants within a plot had open flowers. Similarly, days to maturity was calculated as the number of days required from sowing to the stage of 90% yellow-coloured plants in each plot. Prior to harvesting, the Reglone^®^ (Diquat) was applied to remove excess moisture and prepare the plants for harvesting. For 2017, six randomly selected plants were hand harvested for biomass and yield component measurements, whereas in 2018, three plants were randomly hand harvested from each microplot. Finally, individual plots were harvested within two weeks after desiccation to ensure limited or no shattering loss. These plants were used to determine the total seed weight per plant (g), number of seeds per plant, and biomass per plant (g). All the harvested plants were dried with warm air circulation at 30 to 40 °C for 48 h until a constant dry weight was achieved. Prior to threshing, the whole plant samples were weighed for total biomass, and then the samples were threshed using a rubber belt threshing machine. The harvested seeds were differentiated into three distinct categories such as kabuli, desi, and pea type. The clean seeds obtained from hand harvested plants were used for grain yield measurements (g). Total number of seeds per plant were calculated by using an electronic seed counter (ESC-1; Agriculex Inc., Guelph, ON, Canada). The biomass and seed yield data were used to calculate seed yield and number of seeds per m^2^ plot, and harvest index. The harvest index was calculated by the following formula:

$$Harvest\;index=\left(Total\;seed\;yield÷Total\;biomass\;yield\right)\times 100$$

### 4.4. Genotyping of Chickpea Populations and Data Analysis

The seeds of 381 lines at F_5_ generation as well as 20 wild parents and the cultivated parent were grown in the greenhouse during the fall of 2017 to collect leaf tissue for DNA source and molecular analyses. The seedlings were grown up to four leaves stage to collect the required amount of leaf tissue for DNA analyses. Approximately 150 mg of fresh leaf tissues were carefully collected in microtubes. The collected fresh leaf tissues were then freeze-dried and stored at −80 °C. Later, the samples were sent to the genotyping service laboratory, Freedom Markers in Iowa, USA. Genotyping of the chickpea lines was conducted by a modified genotyping-by-sequencing (GBS) protocol called tunable genotyping-by-sequencing (tGBS) [79]. The genomic DNA from the leaf tissue of 402 lines (381 interspecific lines, 20 wild parents, and 1 cultivated parent) was extracted using the MagAttract 96 DNA Plant Core Kit (QIAGEN; Valencia, CA, USA) following the manufacturer protocol. The DNA samples were normalized using the Qubit dsDNA Broad Range Assay [Thermo Fisher (Waltham, MA, USA)]. In total, 120 ng of DNA from each sample was used for tGBS library preparation according to the tGBS protocol [79]. The tGBS libraries were then sequenced on Life Technologies’ Ion Proton Systems following the Ion PI Hi-Q Sequencing 200 Kit User Guide. Clean reads were aligned to the CDC Frontier reference genome (V1.0) using GSNAP [97], and SNPs were called. All these steps for genotyping of 402 chickpea germplasms were performed by the genotyping service laboratory, Freedom Markers in Iowa, USA.

### 4.5. Statistical Analyses

Statistical analyses were carried out using the R package (3.4.0 version: an open-source statistical software from the www.r-project.org; accessed on 20 May 2019). Prior to analyses, the data were tested for normality using Shapiro–Wilk’s test, and homogeneity of variance was validated using Bartlett’s test. The years and locations were used for descriptive analysis. Mean data from each location were used for calculating phenotypic correlation among the agronomic and yield traits. The SEM was used to calculate the direct and indirect effects of agronomic and yield components on the yield of chickpea in the R program (3.4.0 version). In SEM, the covariance and correlation estimate of the traits allowed a better estimate to determine the direct and indirect effects of the independent variables on yield. The mean values of nine phenotypic traits were used for cluster analysis. The genetic diversity was determined by Euclidean Ward’s method, and the data were standardized before analysis [98]. Cluster visualization was conducted via heatmap using the online tool Clustvis (http://biit.cs.ut.ee/clustvis/, accessed on 1 June 2019).

Analysis of variance (ANOVA) for all agronomic and yield traits from 2018 field trials were performed using the mixed linear model (MLM). All the measured traits were considered as dependent variables. For ANOVA, the lines and locations were considered as fixed effects, while replications were considered as random. The variance components were calculated in R package and used to calculate the broad sense heritability (*H*^2^). The *H*^2^ for each trait was calculated using the following equation:

$$H^{2}=\frac{\sigma^{2}G}{\sigma^{2}G+\sigma^{2}er}\;\;\;\;\;\;\;\;\;\;\;\;\;and\;\;\;\;\;\;\;\;\;\;\;\;\;H^{2}=\frac{\sigma^{2}G}{\sigma^{2}G+\sigma^{2}GE+\sigma^{2}er}$$

where $\sigma^{2}G$, $\sigma^{2}GE$, and $\sigma^{2}er$ indicates the estimates of genotype, genotype-environment, and error variance respectively [99].

### 4.6. Genetic Diversity and Population Structure Analyses

The SNPs used for genetic diversity and population structure analyses were obtained at minor allele frequency (MAF) ≥ 1% using TASSEL 5.2.13 software. To analyze the genetic diversity, similar software (TASSEL 5.2.13) was used for generating the phylogenetic relationship among the chickpea lines. The SNPs data were also used to determine the level of genetic diversity among the parental germplasms. A phylogenetic tree based on the genetic-distance of 381 F_5_ lines plus 20 parents and 1 cultivated (total 401 genotypes) was generated by using the neighbour-joining method with 100 bootstrap replicates [100]. The MEGA X software was used to visualize the phylogenetic tree generated by neighbour-joining method [101].

A total of 14,591 SNP markers with MAF ≥ 1% were used for population structure analysis based on the allele frequency by using the ADMIXTURE software 1.23 [102,103]. To identify the number of *K* inferring the structure of the lines, the ADMIXTURE was set with a predefined *K* value (*K* = 2 to 10) which corresponds to the number of parental population clusters. Each population cluster was run 20 times in order to find out the best *K* value. The optimum number of *K* was calculated using the STRUCTURE SELECTOR (an online visualizing program) by uploading the Q files generated from ADMIXTURE analysis [104].

### 4.7. Genome-Wide Association Study (GWAS)

The mean phenotypic data recorded during 2017 (Saskatoon and Moose Jaw) and 2018 (Limerick and Lucky Lake) were combined with the SNP markers information through genome-wide association analysis to identify significant markers associated with a particular trait. For association analysis, 381 F_5_ lines obtained from 20 interspecific crosses were used. The presence of marker-trait association was calculated by using 5501 polymorphic SNPs with MAF ≥ 5%. Alleles in the F_5_ lines were either inherited from the founder parents (19 wild parents) or the cultivated parent (CDC Leader). Therefore, the homozygous alleles of founder and reference parents were coded as zero and two, respectively, and the heterozygous allele was coded as one for GWAS. The association analysis was performed using the R statistical program using the NAM package [105]. This package was designed to carry out an association analysis suitable for populations grouped in multiple families. The NAM package was developed based on the MLM that consider SNPs and families as cofactors. The MLM calculated the *p*-values and the proportions of variance explained by all the SNPs for a particular trait that controls the genetic background and structure of the population. In this MLM, the heterogeneity of the genetic background was separated as heterozygous alleles that reduces the chance of generating false positive association. After detecting large numbers of associations between markers and desired traits, the false discovery rate (FDR = 0.25) test was applied to declare the significant markers [105]. The FDR test reduced the number of markers associated with the individual trait. Candidate genes were identified on 100 kb region on either side of the significant makers.

## 5. Conclusions and Future Research

The lines derived from interspecific crosses between a cultivated chickpea variety and wild accessions of *Cicer reticulatum* were highly variable for the agronomic and yield traits. The valuable alleles derived from the wild accessions were confirmed by phenotypic and genotypic evaluations. The correlation and path coefficient analyses revealed that seed weight per plant, thousand seed weight, number of seeds per plant, and biomass yield were the most significant yield contributing traits to enhance the seed yield potential of cultivated chickpeas. Cluster analysis based on the agronomic and yield contributing traits categorized the lines into six distinct clusters, which provides the potential for future improvement by crossing the lines among the clusters for yield improvement and resistance to ascochyta blight disease. The heritability estimate showed a range of moderate-to-high values indicating that selection could be made for the traits for further gain in genetic improvement. Genotyping using the SNP markers confirmed the high genetic diversity of the progeny lines. The results of the SNP-based genetic diversity are highly correlated with their pedigree. Association analysis identified SNPs that are significantly associated with early flowering and yield per plant. Overall, our study results revealed the successful development of breeding lines from interspecific crosses between cultivated and *C. reticulatum* which had a greater genetic diversity as well as a significant marker-trait association for important traits.

The findings from this research could be used for selecting the specific interspecific lines with improved traits for use as parents in breeding program. The SNPs associated with the traits can be used to aid the selection of the progeny. The following research areas, which are beyond the scope of the current research, need further investigation. The variable genotype-environment interactions observed in this study need further investigation in a wide range of environmental conditions to identify the most stable genotypes. The selected genotypes with early flowering and high yield could be evaluated in the multi-years and multi-locations experiment. Furthermore, whole genome resequencing could be used for SNP validation and further association analysis which will improve the possibility to identify tightly linked markers or candidate genes for the desired traits.

## Figures and Tables

**Table 1:** Descriptive statistics of 486 F_4_’ lines derived from interspecific crosses of *C. arietinum* and *C. reticulatum*, and CDC Leader for yield and selected yield contributing traits evaluated at two locations (Saskatoon and Moose Jaw) in Saskatchewan in 2017.

Traits	Saskatoon-2017				Moose Jaw-2017			
	F_4_ Lines		CDC Leader		F_4_ Lines		CDC Leader	
	Range	Mean	Range	Mean	Range	Mean	Range	Mean
Days to flowering	47.0–63.0	53.0 (5.00)	49.0–54.0	52.0 (2.00)	43.0–58.0	50.0 (5.00)	50.0–53.0	52.0 (2.00)
Days to maturity	72.0–99.0	89.0 (7.40)	87.0–93.0	90.0 (2.00)	70.0–99.0	88.0 (8.00)	89.0–92.0	91.0 (2.00)
Plant height (cm)	20.0–64.0	31.9 (19.0)	32.0–35.0	33.0 (1.73)	18.0–50.0	28.9 (15.0)	30.0–36.0	32.7 (3.06)
Ascochyta blight score	4.00–8.00	6.22 (1.40)	0.0	0.0	4.00–9.00	5.39 (2.30)	0.0	0.0
Biomass yield per plant (g)	10.5–346	53.1 (99.0)	10.7–20.9	14.6 (5.51)	1.30–98.8	13.5 (88.0)	9.28–15.6	13.1 (3.37)
Number of seeds per plant	1.00–180	43.0 (77.0)	21.0–42.0	33.0 (11.0)	1.00–53.0	18.0 (51.0)	14.0–49.0	27.0 (19.0)
Thousand seed weight (g)	101–695	361 (27.0)	211–250	227 (20.3)	121–453	246 (21.0)	239–268	256 (15.1)
Seed weight per plant (g)	0.10–96.0	10.7 (75.0)	4.44–8.74	7.00 (2.28)	0.10–14.2	4.37 (54.0)	8.86–11.7	10.1 (1.47)
Seed yield (kg/ha)	-	-	-	-	100–6000	1820 (55.0)	2392–2676	2563 (150)
Harvest index	0.02–0.84	0.31 (37.0)	0.48–0.56	0.50 (0.04)	0.01–0.69	0.40 (39.0)	0.35–0.56	0.40 (0.12)

Values in parentheses are the variance of the mean. Dash (-) indicates the traits were not measured for that site.

**Table 2:** Descriptive statistics of 381 F_5_’ lines derived from interspecific crosses of *C. arietinum* and *C. reticulatum* and CDC Leader for yield and selected yield contributing traits evaluated at two different locations (Limerick and Lucky Lake) in Saskatchewan in 2018.

Traits	Limerick-2018				Lucky Lake-2018			
	F_5_ Lines		CDC Leader		F_5_ Lines		CDC Leader	
	Range	Mean	Range	Mean	Range	Mean	Range	Mean
Days to flowering	42.0–57.0	49.0 (4.80)	55.0–57.0	56.0 (1.00)	31.0–53.0	45.0 (5.50)	50.0–54.0	52.0 (2.00)
Days to maturity	82.0–96.0	90.0 (2.70)	88.0–93.0	91.0 (3.00)	75.0–95.0	88.0 (2.90)	89.0–93.0	91.0 (2.00)
Plant height (cm)	22.0–45.0	32.0 (17.0)	34.0–38.0	36.0 (2.08)	16.7–35.0	27.4 (13.0)	28.0–32.0	30.0 (2.00)
Ascochyta blight score	4.00–9.00	6.90 (32.0)	-	-	-	-	-	-
Biomass yield per plant (g)	1.00–37.2	13.9 (19.0)	15.0–18.0	13.0 (3.66)	3.20–57.6	17.5 (17.0)	13.1–19.7	15.8 (3.50)
Number of seeds per plant	2.00–45.0	15.0 (16.0)	44.0–51.0	47.0 (4.00)	3.00–103	26.0 (16.0)	16.0–31.0	21.0 (8.00)
Thousand seed weight (g)	136–467	236 (15.0)	262–281	270 (9.00)	135–576	261 (11.0)	271–358	335 (57.0)
Seed weight per plant (g)	0.40–10.6	3.49 (32.0)	11.8–14.3	12.8 (1.34)	1.70–14.6	6.73 (30.0)	7.17–11.1	8.70 (2.01)
Seed yield (kg/ha)	20–4400	1460 (28.0)	2623–2807	2703 (95)	100–3900	1740 (28.0)	3302–3533	3395 (122)
Harvest index	0.02–0.71	0.27 (29.0)	0.77–0.80	0.78 (0.01)	0.13–0.66	0.38 (18.0)	0.54–0.56	0.50 (0.01)

Values in parentheses are the variance of the mean. Dash (-) indicated that no ascochyta blight disease was observed for that site.

**Table 3:** Pearson correlation coefficients among the yield and yield contributing traits of 486 F_4_’ lines derived from interspecific crosses of *C. arietinum* and *C. reticulatum* evaluated in Saskatoon, Saskatchewan in 2017.

Traits	DTM	PH	ABS	BY	NSPP	TSW	HI	SWPP
DTF	0.02 ^ns^	0.04 ^ns^	−0.04 ^ns^	0.01 **	−0.13 *	0.08 ^ns^	−0.28 ***	−0.11 *
DTM		−0.03 ^ns^	0.07 ^ns^	−0.07 ^ns^	−0.15 **	0.11 *	−0.25 ***	−0.13 *
PH			−0.18 ***	0.63 ***	0.50 ***	0.16 ***	−0.18 ***	0.51 ***
ABS				−0.17 ***	−0.22 ***	0.01 ^ns^	−0.05 ^ns^	−0.24 ***
BY					0.78 ***	0.26 ***	−0.20 ***	0.82 ***
NSPP						−0.05 ^ns^	0.21 ***	0.95 ***
TSW							−0.24 ***	0.09 ^ns^
HI								0.22 ***

Different evaluated traits are DTF: days to flowering; DTM: days to maturity; PH: plant height (cm); ABS: ascochyta blight disease score; BY: biomass yield per plant (g); NSPP: number of seeds per plant (g); TSW: thousand seed weight (g); HI: harvest index; SWPP: seed weight per plant (g). ^ns^, *, ** and ***: non-significant, significant at *p* < 0.05, *p* < 0.01, and *p* < 0.001.

**Table 4:** Pearson correlation coefficients among the yield and yield contributing traits of 486 F_4_’ lines derived from interspecific crosses of *C. arietinum* and *C. reticulatum* evaluated in Moose Jaw, Saskatchewan in 2017.

Traits	DTM	PH	ABS	BY	SWPP	NSPP	TSW	HI	SY
DTF	0.19 ***	0.21 ***	0.03 ^ns^	0.15 ***	0.05 ^ns^	0.01 ^ns^	0.14 **	−0.18 ***	0.08 ^ns^
DTM		0.12 *	0.09 ^ns^	0.29 ***	0.07 ^ns^	0.07 ^ns^	0.08 ^ns^	−0.35 ***	0.10 *
PH			−0.01 ^ns^	0.33 ***	0.29 ***	0.23 ***	0.16 ***	−0.03 ^ns^	0.28 ***
ABS				0.07 ^ns^	0.04 ^ns^	0.04 ^ns^	0.03 ^ns^	−0.06 ^ns^	0.02 ^ns^
BY					0.80 ***	0.75 ***	0.22 ***	−0.21 ***	0.76 ***
SWPP						0.90 ***	0.37 ***	0.35 ***	0.99 ***
NSPP							−0.04 ^ns^	0.27 ***	0.88 ***
TSW								0.26 ***	0.36 ***
HI									0.33 ***

Different evaluated traits are DTF: days to flowering; DTM: days to maturity; PH: plant height (cm); ABS: ascochyta blight disease score; BY: biomass yield per plant (g); SWPP: seed weight per plant (g); NSPP: number of seeds per plant; TSW: thousand seed weight (g); HI: harvest index; SY: seed yield (kg/ha). ^ns^, *, ** and ***: non-significant, significant at *p* < 0.05, *p* < 0.01, and *p* < 0.001.

**Table 5:** Pearson correlation coefficients among the yield and yield contributing traits of 381 F_5_’ lines derived from interspecific crosses of *C. arietinum* and *C. reticulatum* evaluated in Limerick, Saskatchewan in 2018.

Traits	DTM	PH	ABS	BY	SWPP	NSPP	TSW	HI	SY
DTF	0.28 ***	0.23 ***	−0.16 **	0.22 ***	0.01 ^ns^	−0.01 ^ns^	0.11 **	−0.15 ***	0.00 ^ns^
DTM		0.29 ***	0.12 **	0.21 ***	−0.07 ^ns^	−0.06 ^ns^	0.10 *	−0.15 ***	−0.07 ^ns^
PH			−0.03 ^ns^	0.37 ***	−0.05 ^ns^	−0.05 ^ns^	0.03 ^ns^	−0.21 ***	−0.04 ^ns^
ABS				−0.33 ***	−0.07 ^ns^	−0.01 ^ns^	−0.15 ***	0.13 *	−0.06 ^ns^
BY					0.07 ^ns^	0.05 ^ns^	0.11 *	−0.33 ***	0.08 ^ns^
SWPP						0.92 ***	0.17 ***	0.69 ***	0.99 ***
NSPP							−0.13 **	0.69 ***	0.91 ***
TSW								0.04 ^ns^	0.18 ***
HI									0.67 ***

Different traits are DTF: days to flowering; DTM: days to maturity; PH: plant height (cm); ABS: ascochyta blight disease score; BY: biomass yield per plant (g); SWPP: seed weight per plant (g); NSPP: number of seeds per plant; TSW: thousand seed weight (g); HI: harvest index; SY: seed yield (kg/ha). ^ns^, *, ** and ***: non-significant, significant at *p* < 0.05, *p* < 0.01, and *p* < 0.001.

**Table 6:** Pearson correlation coefficients among the yield and yield contributing traits of 381 F_5_’ lines derived from interspecific crosses of *C. arietinum* and *C. reticulatum* evaluated in Lucky Lake, Saskatchewan in 2018.

Traits	DTM	PH	BY	SWPP	NSPP	TSW	HI	SY
DTF	0.53 ***	0.28 ***	0.25 ***	0.01 ^ns^	0.04 ^ns^	0.17 ***	−0.14 ***	0.03 ^ns^
DTM		0.31 ***	0.37 ***	−0.07 ^ns^	0.14 ***	0.16 ***	−0.16 ***	0.05 ^ns^
PH			0.18 ***	0.06 ^ns^	0.02 ^ns^	0.16 ***	−0.04 ^ns^	0.13 **
BY				0.03 ^ns^	0.80 ***	0.17 ***	−0.20 ***	0.34 ^ns^
SWPP					0.27 ***	0.16 ***	0.39 ***	0.54 ***
NSPP						−0.16 ***	0.48 ***	0.42 ***
TSW							0.30 ***	0.31 ***
HI								0.57 ***

Different traits are DTF: days to flowering; DTM: days to maturity; PH: plant height (cm); BY: biomass yield per plant (g); SWPP: seed weight per plant (g); NSPP: number of seeds per plant; TSW: thousand seed weight (g); HI: harvest index; SY: seed yield (kg/ha). ^ns^, ** and ***: non-significant, significant at *p* < 0.01, and *p* < 0.001.

**Table 7:** Direct and indirect effects of yield contributing traits on seed yield of F_4_’ lines evaluated in Saskatoon and Moose Jaw in 2017.

Pathway	Direct Effect			
	Saskatoon-2017		Moose Jaw-2017	
	Standardized Estimates	Standard Error	Standardized Estimates	Standard Error
BY→ NSPP	0.85 ***	0.03	0.17 ***	0.00
BY→ SWPP	0.11 **	0.01	0.01 ^ns^	0.00
BY→ HI	−0.98 ***	0.00	−0.63 ***	0.00
TSW→ NSPP	−0.27 ***	0.00	−0.01 ^ns^	0.01
TSW→ SWPP	0.10 **	0.00	0.36 ***	0.00
TSW→ HI	−0.06 ^ns^	0.00	0.24 ***	0.00
NSPP→ SWPP	0.87 ***	0.01	0.91 ***	0.00
NSPP→ HI	0.01 ^ns^	0.00	0.48 ***	0.00
SWPP→ HI	0.98 ***	0.00	0.07 ^ns^	0.01
	**Indirect effect**			
BY→ HI (Through NSPP)	0.00	-	0.08	-
BY→ HI (Through SWPP)	0.11	-	0.00	-
TSW→ HI (Through NSPP)	−0.00	-	−0.00	-
TSW→ HI (Through SWPP)	−0.01	-	0.03	-
NSPP→ HI (Through SWPP)	0.00	-	0.43	-

Pathway of different traits are BY: biomass yield per plant (g); SWPP: seed weight per plant; NSPP: number of seeds per plant; TSW: thousand seed weight (g); HI: harvest index. The variable at the tail affects the variable at the head. ^ns^, **, and ***: non-significant, significant at *p* < 0.01, and *p* < 0.001.

**Table 8:** Direct and indirect effects of yield contributing traits on seed yield of F_5′_ lines evaluated in Limerick and Lucky Lake in 2018.

Pathway	Direct Effect			
	Limerick-2018		Lucky Lake-2018	
	Standardized Estimates	Standard Error	Standardized Estimates	Standard Error
BY→ NSPP	0.06 ^ns^	0.07	0.75 ***	0.06
BY→ SWPP	−0.00 ^ns^	0.00	0.01 ^ns^	0.03
BY→ HI	−0.38 ***	0.00	−0.59 ***	0.00
TSW→ NSPP	−0.14 **	0.01	−0.22 ***	0.01
TSW→ SWPP	0.30 ***	0.00	0.07 ^ns^	0.00
TSW→ HI	0.14 **	0.00	0.50 ***	0.00
NSPP→ SWPP	0.96 ***	0.00	0.02 ^ns^	0.02
NSPP→ HI	0.62 ***	0.00	0.98 ***	0.00
SWPP→ HI	0.13 *	0.01	0.06 ^ns^	0.00
	**Indirect effect**			
BY→ HI (Through NSPP)	0.04	-	0.74	-
BY→ HI (Through SWPP)	−0.00	-	0.00	-
TSW→ HI (Through NSPP)	−0.09	-	0.22	-
TSW→ HI (Through SWPP)	0.04	-	0.00	-
NSPP→ HI (Through SWPP)	0.12	-	0.00	-

Pathway of different traits are BY: biomass yield per plant (g); SWPP: seed weight per plant; NSPP: number of seeds per plant; TSW: thousand seed weight (g); HI: harvest index. The variable at the tail affects the variable at the head. ^ns^, *,**, and ***: non-significant, significant at *p* < 0.05, *p* < 0.01, and *p* < 0.001.

**Table 9:** Analysis of variance (ANOVA) and broad sense heritability estimates (*H*^2^) of the chickpea lines (F_5_’ generation) for the yield and yield contributing traits evaluated at two locations (Limerick and Lucky Lake), Saskatchewan in 2018.

Traits	*F* Values of the Effects			*H* ^2^
	G	E	G × E	
Days to flowering	6.36 ***	919 ***	1.03 ^ns^	0.54
Days to maturity	4.39 ***	262 ***	1.15 *	0.35
Plant height (cm)	2.06 ***	479 ***	0.93 ^ns^	0.15
Biomass weight per plant (g)	1.93 ***	39.8 ***	1.03 ^ns^	0.14
Number of seeds per plant	2.78 ***	681 ***	1.56 ***	0.18
Thousand seed weight (g)	6.47 ***	263 ***	6.16 ***	0.08
Seed weight per plant (g)	3.04 ***	710 ***	1.43 ***	0.45

G: Genotype; E: Environment; G x E: Genotype and Environment interaction. ^ns^, *, and ***: non-significant, significant at *p* < 0.05, and *p* < 0.001.

**Table 10:** Mean and standard deviation of 6 clusters for yield and yield contributing traits toward genetic divergence in 381 chickpea lines at the F_5_’ generation.

Traits	Cluster-I (67)	Cluster-II (104)	Cluster-III (68)	Cluster-IV (72)	Cluster-V (42)	Cluster-VI (28)
Days to flowering	47.0 ± 2.0	47.0 ± 3.0	46.0 ± 2.0	50.0 ± 3.0	51.0 ± 2.0	45.0 ± 2.0
Days to maturity	88.0 ± 2.0	90.0 ± 2.0	87.0 ± 2.0	91.0 ± 2.0	89.0 ± 2.0	87.0 ± 1.0
Plant height (cm)	29.2 ± 3.6	28.8 ± 2.4	28.2 ± 2.7	33.3 ± 2.4	28.9 ± 2.1	29.0 ± 2.4
Ascochyta blight score	6.64 ± 1.6	7.81 ± 0.7	8.00 ± 0.7	6.83 ± 1.4	5.48 ± 1.5	4.65 ± 0.9
Biomass yield per plant (g)	17.6 ± 4.0	14.7 ± 3.3	10.8 ± 5.5	20.5 ± 4.3	16.4 ± 3.8	13.6 ± 4.3
Number of seeds per plant	28.0 ± 7.0	19.0 ± 6.0	17.0 ± 6.0	22.0 ± 6.0	21.0 ± 5.0	18.0 ± 4.0
Thousand seed weight (g)	253 ± 28	238 ± 31	234 ± 34	268 ± 43	245 ± 31	260 ± 45
Seed weight per plant (g)	6.87 ± 1.4	4.20 ± 1.1	5.32 ± 1.5	4.90 ± 1.4	4.65 ± 1.1	4.96 ± 1.4
Seed yield (kg/ha)	2310 ± 394	1332 ± 384	1360 ± 432	1680 ± 394	1598 ± 300	1650 ± 528

Values in parentheses are the number of lines in each cluster. “±values” indicates the standard deviations.

**Table 11:** List of significant SNPs from genome-wide association analysis for the traits evaluated in Saskatoon and Moose Jaw in 2017.

Traits	Field Sites	Chromosome	Most Significant SNP	Number of SNPs	−log_10_ *p* Value
Days to flowering	Combined	VI	Ca6_V1_P-46744160	1	5.24
Biomass	Combined	III	Ca3_V1_P-31624927	1	6.26
Number of seeds per plant	Saskatoon	II	Ca2_V1_P-33400910	2	10.2
		IV	Ca4_V1_P-27008886	2	6.63
		V	Ca5_V1_P-28287194	1	6.04
	Moose Jaw	VI	Ca6_V1_P-22032893	2	5.09
Thousand seed weight (g)	Moose Jaw	I	Ca1_V1_P-710760	1	5.30
		IV	Ca4_V1_P-9707182	5	6.60
Seed weight per plant (g)	Saskatoon	I	Ca1_V1_P-25733193	1	5.01
		II	Ca2_V1_P-30049933	2	6.32
		IV	Ca4_V1_P-40127929	1	5.12
		V	Ca5_V1_P-41263716	3	6.94
		VI	Ca6_V1_P-41147990	3	9.32
		VII	Ca7_V1_P-45085937	3	5.19

Ca = *Cicer arietinum*; V1 = Version 1; P = Position on chromosome in base pairs.

**Table 12:** List of significant SNPs from genome-wide association analysis of the traits evaluated in Limerick and Lucky Lake in 2018.

Traits	Field Sites	Chromosome	Most Significant SNP	Number of SNPs	−log_10_ *p* Value
Days to flowering	Combined	IV	Ca4_V1_P-13022400	9	10.4
Days to maturity	Lucky Lake	VIII	Ca8_V1_P-957257	1	5.08
Biomass	Combined	VII	Ca7_V1_P-34285390	2	12.1
Number of seeds per plant	Lucky Lake	II	Ca2_V1_P-15088105	1	5.02
		III	Ca3_V1_P-14460088	1	5.14
		VII	Ca7_V1_P-34285390	2	6.79
		VIII	Ca8_V1_P-310610	1	8.95
Thousand seed weight (g)	Limerick	I	Ca1_V1_P-14313744	1	6.54
		II	Ca2_V1_P-17609263	2	6.28
		V	Ca5_V1_P-32629686	1	5.32
	Lucky Lake	V	Ca5_V1_P-29349635	1	5.23
		VI	Ca6_V1_P-16130634	1	11.2

Ca = *Cicer arietinum*; V1 = Version 1; P = Position on chromosome in base pairs.

**Table 13:** Candidate gene annotations for the studied traits and their position on the chickpea genome.

Gene Id	Chromosome	Start	End	Description	Gene Function	Reference
Related to flowering						
Ca_TIC	IV	13836536	13844034	protein (tic)	Early flowering	[26]
Ca_GA20OX2	IV	13002067	13004480	gibberellin 20 oxidase 2	Associated with flowering time	[27]
Ca_PCL1	VI	54242622	54245220	transcription factor PCL1-like	Associated with flowering time	[28]
Related to yield						
Ca_10265	II	32585905	32594820	Protein kinase	Regulates photophosp-horylation activity	[29]
Ca_10221	II	33011956	33016412	Protein kinase	Regulates photophosp-horylation activity	[29]
Ca_10204	II	33172919	33174836	Plastocyanin-like	Involved in electrons to photosystem I	[30]
Ca_14921	IV	39853369	39854663	Photosystem I PsaG/PsaK protein	Involved in photosynthesis	[31]

## Data Availability

All data are available through corresponding author.
